# Low-grade myofibroblastic sarcoma of the proximal femur: A case report and literature review

**DOI:** 10.1097/MD.0000000000031715

**Published:** 2022-11-11

**Authors:** Guanying Gao, Yuhao Liu, Yingfang Ao, Jianquan Wang, Yan Xu

**Affiliations:** a Institute of Sports Medicine, Beijing Key Laboratory of Sports Injuries, Peking University Third Hospital, Beijing, China.

**Keywords:** Low-grade myofibroblastic sarcoma, proximal femur, hip, arthroscopy

## Abstract

Low-grade myofibroblastic sarcoma (LGMS) is an atypical type of tumor composed of myofibroblasts. LGMS in the femoral head neck junction is extremely rare and no case treated by hip arthroscopy was reported.

**Patient concerns:**

We reported a case of LGMS in the femoral head neck junction treated by hip arthroscopy. A 30-year-old female was admitted to our hospital with discomfort and pain after left hip sprained one year prior. Physical examination revealed swelling of the left hip and magnetic resonance images showed a soft tissue mass in the femoral head neck junction.

**Diagnosis:**

Via microscopy of pathological specimens, spindle cell proliferative lesions, atypia of some cells, and mitotic figures/pathological mitotic figures of some cells were observed. Immunohistochemistry revealed positive for smooth muscle actin, focally positive for CD34 and CD68, while negative for S-100, desmin, and anaplastic lymphoma kinase. The imaging, histomorphological and immunohistochemical features suggested a final diagnosis of LGMS of the proximal femur.

**Interventions:**

This patient underwent hip arthroscopy for excision of the soft tissue mass.

**Outcomes:**

The clinical and imaging follow-up at 6 months postoperatively showed that surgery had achieved good clinical outcomes.

**Lessons:**

To the best of our knowledge, this is the first case report of LGMS in the femoral head neck junction treated by hip arthroscopy. Beyond the present case, other 120 cases from 58 literatures (1998–2022) are reviewed and discussed. The age of LGMS patients ranged from 11 months to 77 years and the male-to-female ratio was approximately 1.28:1. The location distribution of previously reported LGMS cases and the present case was as follows: Head&neck (45.90%), trunk (30.33%), and extremity (23.77%). Hip arthroscopic excision of LGMS may achieve relatively good clinical outcomes.

## 1. Introduction

Low-grade myofibroblastic sarcoma (LGMS) was firstly described by Mentzel et al^[1]^, which represents an atypical and extremely rare type of tumor composed of myofibroblasts. It was first classified as a new group of soft tissue and bone tumors by the WHO in 2002, and this classification was maintained in 2020.^[2]^ LGMS has been reported to occur in deep soft tissues with predilection for the head and neck.^[3]^ However, according to previous study by Kim et al,^[4]^ the incidence of LGMS in the extremities or trunk may be higher than expected. Based on the rarity of this condition, this study aims to introduce a case report of LGMS in proximal femur and to perform a review of relevant literature.

## 2. Case presentation

A 30-year-old female was admitted to our hospital with discomfort and pain after left hip sprained 1 year prior. The pain was progressive in nature. Physical examination revealed swelling of the left hip with tenderness in the greater trochanter, groin area, and posterior buttock. This patient had severe limited hip range of motion and had to walk on crutches. No significant personal or family history was present.

**Figure 1.**
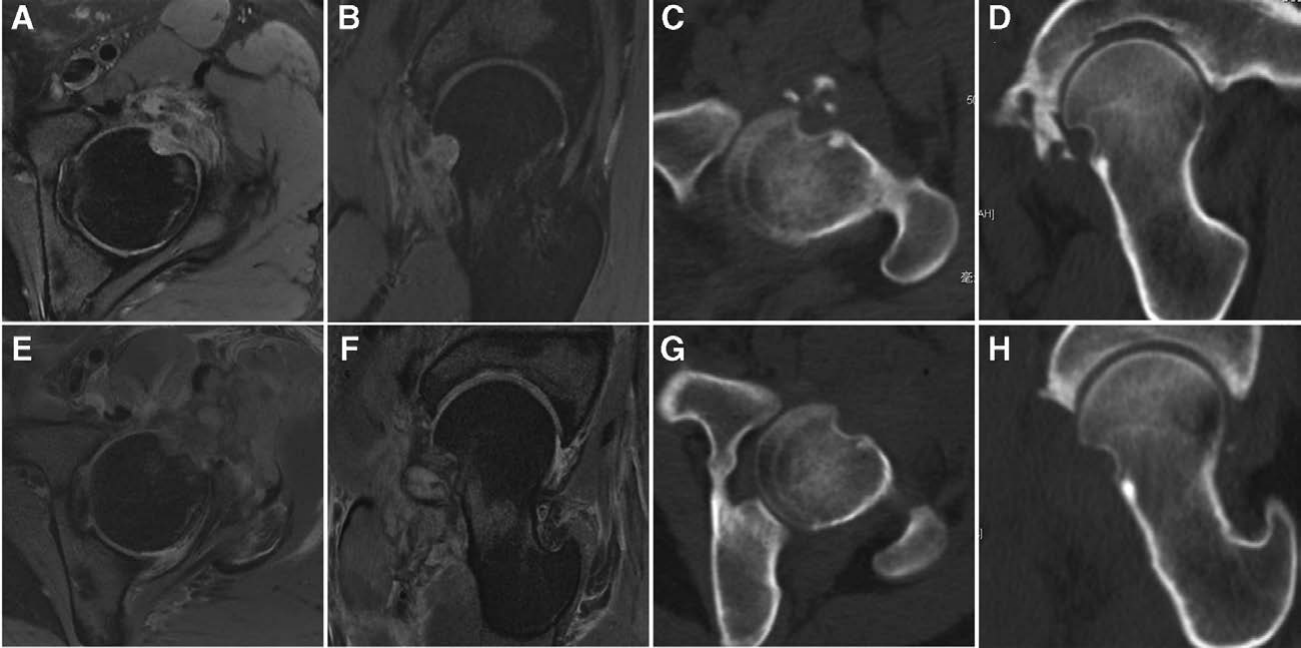
(A, B) Preoperative axial and oblique sagittal MRI showed soft tissue mass in the femoral head neck junction. (C, D) Preoperative axial and oblique sagittal CT showed adjacent bone defect and ossification. (E, F) Postoperative axial and oblique sagittal MRI showed total excision of the mass. (G, H) Postoperative axial and oblique sagittal CT showed total excision of the mass. CT = computed tomography, MRI = magnetic resonance images.

Magnetic resonance images (MRI) showed a soft tissue mass in the anterolateral femoral head neck junction in the left hip (Fig. 1A and B). The mass appeared heterogenously hyperintence in T2-weighted image. Edema of surrounding tissue and adjacent bone defect in femoral head neck junction were observed. Computed tomography (CT) showed ossification in adjacent acetabulum (Fig. 1C and D).

This patient underwent hip arthroscopy for excision of the mass. Outside-in approach was used because of anterior ossification. The soft tissue mass, adjacent bone tissue, hyperplastic synovium, and ossification in the acetabulum were debrided. The range of motion returned normal after surgery, just like the contralateral hip. Two pathological specimens were taken during the operation, which were acetabulum ossification and soft tissue mass. The bony tissue of the left acetabulum contained part of dense connective tissue, in which a few proliferative spindle cells could be seen and the shape was mild. In the left hip soft tissue mass, spindle cell proliferative lesions were observed. Some cells showed atypia. Some cells had mitotic figures, and occasionally pathological mitotic figures. Tumor borders were difficult to identify. The soft tissue mass was sent for immunohistochemistry, which revealed positive for smooth muscle actin (SMA), focally positive for CD34 and CD68, while negative for S-100, Desmin, P53, nuclear β-catenin, ALK1, SOX10, MUC4, and STAT6.

**Figure 2.**
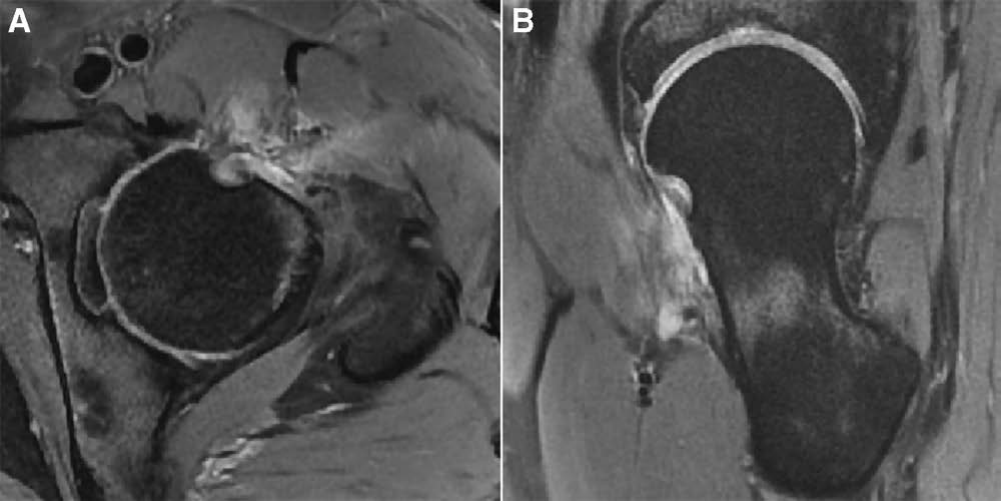
(A, B) Axial and oblique sagittal MRI 6 months after surgery showed no local recurrence. MRI = magnetic resonance image.

The imaging, histomorphological, and immunohistochemical features suggested a final diagnosis of LGMS of the proximal femur. Postoperative CT and MRI were conducted 1 day after surgery and revealed that the tumor was completely removed (Fig. 1E–H). Adjuvant chemotherapy or radiotherapy were not used for this patient. The patient underwent MRI follow-up 6 months after surgery and MRI showed no local recurrence (Fig. 2). Modified Harris Hip Score and visual analog scale for pain improved from 60 and 5 preoperatively to 78 and 1 postoperatively 6 months after surgery.

The patient had provided informed consent for publication of the case, and the study protocol was approved by Medical Ethics Committee of Peking University Third Hospital.

## 3. Discussion

**Figure 3.**
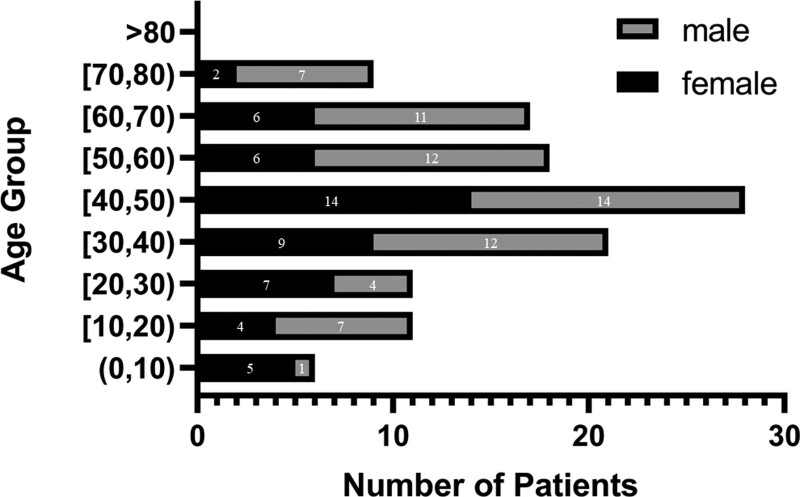
Age and gender distribution of patient populations. According to the reported cases, LGMS patients were mostly among 40- to 49-year-olds. LGMS = low-grade myofibroblastic sarcoma.

Two electronic databases were searched: PubMed (1966-), Web of Science (1900-), with the key words “low-grade myofibroblastic sarcoma” and its synonyms. Non-English literature and inaccessible literature were excluded. We analyzed 120 cases from 58 literatures. With this present case, we have a basic understanding for the information and characteristics of the LGMS patient population (Table 1). Among the 121 reported LGMS patients, the age of them ranged from 11 months to 77 years (mean, 42.45; median, 44). The male-to-female ratio was approximately 1.28:1 (68 males versus 53 females). The detailed age and gender distribution are shown in Figure 3.

**Table 1:** Details of included articles: information and study sample characteristics.

Authors	No. of cases	Age mean	Sex (M/F)	Site	Treatment	Clinical outcome
Mentzel et al.^[1]^	18	42	11/7	Head&neck 5	S 12	NR 8
				Trunk 6	S, RT 2	R 3
				Extremity 7	S, CTX 1	NA 7
					S, RT, CTX 1	
					NA 2	
Montgomery et al.^[5]^	10	54.4	8/2	Head&neck 4	S 7	R 5
				Trunk 3	S, RT 2	NR 4
				Extremity 3	NA 1	NA 1
Chang et al.^[6]^	1	28	0/1	Head&neck 1	S 1	R 1
Roth et al.^[7]^	1	46	0/1	Trunk 1	S 1	NR 1
San Miguel et al.^[8]^	1	51	0/1	Extremity 1	S 1	NR 1
Morgan et al.^[9]^	1	46	0/1	Trunk 1	S 1	NR 1
Tajima et al.^[10]^	1	64	0/1	Trunk 1	S 1	NA 1
Laco et al.^[11]^	1	24	0/1	Head&neck 1	S 1	NR 1
Takahama et al.^[12]^	1	42	1/0	Head&neck 1	RT, CTX 1	DOD 1
Meng et al.^[13]^	14	30.5	9/5	Head&neck 6	S 8	NR 8
				Trunk 5	S, CTX 4	R 5
				Extremity 3	S, RT 2	NA 1
Coyne et al.^[14]^	1	44	1/0	Head&neck 1	S 1	NR 1
Meng et al.^[15]^	3	34	3/0	Head&neck 3	S, RT 3	R 3
Jay et al.^[16]^	1	41	1/0	Head&neck 1	S 1	NR 1
Eisenstat et al.^[17]^	1	5	0/1	Trunk 1	sudden death	NA 1
Agaimy et al.^[18]^	2	60-70	0/2	Trunk 2	S 2	R 2
Nagata et al.^[19]^	1	36	1/0	Extremity 1	S 1	NR 1
Morii et al.^[20]^	1	46	0/1	Trunk 1	S 1	NR 1
Demarosi et al.^[21]^	2	56	1/1	Head&neck 2	S 2	NR 2
Niedzielska et al.^[22]^	1	54	1/0	Head&neck 1	S 1	NR 1
Arora et al.^[23]^	1	38	0/1	Extremity 1	S 1	NR 1
Humphries et al.^[24]^	1	15	0/1	Trunk 1	S 1	NA 1
Montebugnoli et al.^[25]^	1	37	1/0	Head&neck 1	S 1	NR 1
Miyazawa et al.^[26]^	1	65	1/0	Trunk 1	S 1	NR 1
Ni et al.^[27]^	1	41	0/1	Head&neck 1	S 1	NR 1
Yamada et al.^[28]^	1	73	1/0	Head&neck 1	S 1	NR 1
Khosla et al.^[29]^	1	55	1/0	Head&neck 1	S, RT 1	NR 1
Murakami et al.^[30]^	1	24	0/1	Trunk 1	S 1	NR 1
Saito et al.^[31]^	1	50	0/1	Extremity 1	S 1	NR 1
Cai et al (2013)^[32]^	2	30.5	2/0	Head&neck 2	S 2	NR 2
Oylumlu et al.^[33]^	1	36	1/0	Trunk 1	S, CTX 1	Cardiac metastasis
Kordač et al.^[34]^	1	40	1/0	Head&neck 1	S 1	NR 1
Qiu et al.^[35]^	2	37	1/1	Head&neck 2	S 2	NR 1 R 1
Han et al.^[36]^	1	26	1/0	Head&neck 1	S 1	NR 1
Myong et al.^[37]^	1	61	0/1	Trunk 1	S 1	NR 1
Maruyama et al.^[38]^	1	43	0/1	Head&neck 1	S 1	NR 1
Hadjigeorgiou et al.^[39]^	1	55	1/0	Trunk 1	S 1	NR 1
Zhang et al.^[40]^	1	2	0/1	Head&neck 1	S 1	NR 1
Niu et al. ^[41]^	1	51	0/1	Trunk 1	S 1	DOD 1
Mikami et al.^[42]^	1	38	0/1	Head&neck 1	S 1	NR 1
Peng et al.^[43]^	1	44	0/1	Trunk 1	S, CTX 1	NR 1
Taweevisit et al.^[44]^	1	66	1/0	Head&neck 1	S, RT 1	NR 1
Ghosh et al^[45]^	2	36.5	1/1	Head&neck 2	S 2	R 2
Wu et al^[46]^	1	30	1/0	Head&neck and Trunk	supportive treatment	NA 1
Hou et al^[47]^	1	24	0/1	Extremity 1	S 1	NA 1
Bai et al^[48]^	1	74	1/0	Head&neck 1	S 1	NR 1
Scardina et al^[49]^	1	62	0/1	Trunk 1	S 1	R 1
Nair et al.^[50]^	1	63	1/0	Head&neck 1	S 1	NR 1
Mulay et al. ^[51]^	1	48	1/0	Head&neck 1	S, RT 1	NR 1
Tang et al.^[52]^	1	11 month	0/1	Head&neck 1	S 1	NR 1
Kuo et al.^[53]^	1	77	1/0	Trunk 1	S 1	NR 1
Yonezawa et al.^[3]^	1	69	0/1	Head&neck 1	S 1	NR 1
Jayasooriya et al.^[54]^	3	10.7	1/2	Head&neck 3	S 3	NR 2
						NA 1
Padmawar et al.^[55]^	1	13	0/1	Head&neck 1	S 1	NR 1
Kim et al.^[4]^	15	50.8	8/7	Head&neck 1	S 15	NR 13
				Trunk 4		R 2
				Extremity 10		
Emanuelli et al.^[56]^	1	11	1/0	Head&neck 1	S 1	NR 1
Gonçalves et al.^[57]^	1	27	1/0	Head&neck 1	S 1	NR 1
Concepcion-Torio et al.^[58]^	1	38	1/0	Head&neck 1	RT 1	R 1
Lin et al.^[59]^	1	45	1/0	Trunk 1	S, CTX 1	DOD 1
Present case	1	30	0/1	Extremity 1	S 1	NR for now

CTX = chemotherapy, DOD = death of disease, NA = not available, NR = no recurrence, R = recurrence, S = surgery, RT = radiotherapy.

We classified locations of LGMS into 3 groups: Head&neck (56 cases, 45.90%), Trunk (37 cases, 30.33%), and Extremity (29 cases, 23.77%). It should be noted that one of the patients had LGMS which occurred in multiple organs, including the diaphragmatic pleura and head and neck region.^[46]^ The case was counted into Head&neck group and Trunk group, respectively. In addition, several new primary sites of LGMS have been reported in recent years, including limbus,^[51]^ orbit,^[52,58]^ and multiorgan.^[46]^

LGMS was reported to be prone to local recurrence rather than distant metastasis.^[4]^ And the data we collected support this conclusion. Notably, 2 cases of cardiac metastasis of LGMS were reported, both of which resulted in the eventual death of the patient.^[17,33]^ Among the 121 patients with LGMS, the number of *No Recurrence* cases during the follow-up was 76 (62.81%), which suggests that LGMS patients after proper treatment generally have good prognosis. And Chan et al^[60]^ reported that the 5-year overall survival rate of LGMS was 71.6% and the disease-specific survival rate was 76.3%, which is consistent with our conclusion.

The most common treatment for LGMS was surgical excision/wide excision. Some clinicians used chemotherapy or radiotherapy as adjuvant treatment strategies, while most were more concerned about whether the tumor is completely removed. Based on the data we collected, once diagnosed properly and excised wide enough, LGMS is less likely to recur. A quantitative study by Xu et al^[61]^ supports our observations. They reported that age greater than 60 years, positive nodal status, and no surgical treatment were independent prognostic factors for patients with LGMS, whereas chemotherapy and radiation treatment were not. However, some authors reported that LGMS occurring in the Head&neck may not obtain adequate resection margins due to surgical limitations.^[21,32]^ Recently, Lin et al^[59]^ reported that Apatinib functioned as an effective treatment of LGMS via potential VEGFR-PI3K/Akt signaling pathway.

LGMS is an atypical type of tumor composed of myofibroblasts, which results in the strong expression of Vimentin.^[62]^ Therefore, immunohistochemistry can be used for diagnosis and differential identification. Gonçalves et al^[57]^ reported that in their literature review consisting of 30 studies, 26 and 10 of them used α-SMA (alpha-SMA) and muscle-specific actin as myogenic marker, respectively. In our present case, immunohistochemistry revealed positive for SMA, suggesting the tumor cells were derived from myofibroblasts. Also, it’s well known that S-100 protein is a specific biomarker for schwannoma and malignant peripheral nerve sheath tumor.^[11]^ S-100 should be negative in LGMS, which is consistent with our present case. In addition, immunohistochemistry revealed negative for anaplastic lymphoma kinase, helping to differentiate LGMS from inflammatory myofibroblastic tumor.^[63]^ Inflammatory myofibroblastic tumor is a type of tumor similar to LGMS because of their morphologic similarity and the overlapping immunophenotype.^[64]^

## 4. Conclusion

To the best of our knowledge, this is the first case report of LGMS in the femoral head neck junction treated by hip arthroscopy. Beyond the present case, other 120 cases from 58 literatures (1998–2022) are reviewed and discussed. The age of LGMS patients ranged from 11 months to 77 years and the male-to-female ratio was approximately 1.28:1. The location distribution of previously reported LGMS cases and the present case was as follows: head&neck (45.90%), trunk (30.33%), and extremity (23.77%). Wide excision with clear margins may achieve relatively good clinical outcomes.

## Author contributions

**Conceptualization:** Yan Xu, Jianquan Wang, Yingfang Ao, Guanying Gao.

**Data curation:** Guanying Gao, Yuhao Liu.

**Writing—original draft:** Guanying Gao, Yuhao Liu.

**Writing—review and editing:** Yan Xu, Jianquan Wang, Yingfang Ao, Guanying Gao.
